# Trends in mortality from Alzheimer’s disease in Brazil, 2000-2019

**DOI:** 10.1590/S2237-96222023000200002

**Published:** 2023-05-12

**Authors:** Mayara Paschalidis, Thais Cláudia Roma de Oliveira Konstantyner, Sharon Sanz Simon, Camila Bertini Martins

**Affiliations:** 1 Escola Paulista de Medicina, Universidade Federal de São Paulo, São Paulo, SP, Brazil; 2 Columbia University Medical Center, New York, NY, United States

**Keywords:** Alzheimer Disease, Mental Health, Public Health, Time Series Studies, Mortality, Mortality Registries, Enfermedad de Alzheimer, Salud Mental, Salud Pública, Estudios de Series Temporales, Mortalidad, Registros de Mortalidad, Doença de Alzheimer, Saúde Mental, Saúde Pública, Estudos de Séries Temporais, Mortalidade, Registros de Mortalidade

## Abstract

**Objective:**

to analyze trends in mortality rates due to Alzheimer’s disease in Brazil and its macro-regions by age and sex, from 2000 to 2019.

**Methods:**

this was a time-series study on mortality from Alzheimer’s disease in Brazil and its macro-regions by age and sex; data were obtained from the Mortality Information System; a Prais-Winsten model was used to analyze trends.

**Results:**

there were 211,658 deaths in the period analyzed, with an increasing trend in Alzheimer’s disease mortality in Brazil in elderly people aged 60-69 years (APC = 4.3; 95%CI 2.9;5.9), 70-79 years (APC = 8.1; 95%CI 4.8;11.5) and ≥ 80 years (APC = 11.3; 95%CI 8.1;14.6) and in all macro-regions, age groups and sexes.

**Conclusion:**

Brazil and all its macro-regions showed a rising trend in Alzheimer’s disease mortality rates, following the global trend.

## Resumo

**Objetivo:**

analisar as tendências das taxas de mortalidade por doença de Alzheimer no Brasil e nas suas macrorregiões, por faixa etária e sexo, no período de 2000 a 2019.

**Métodos:**

estudo de séries temporais sobre mortalidade por doença de Alzheimer no Brasil e suas macrorregiões por faixa etária e sexo; os dados foram extraídos do Sistema de Informação sobre Mortalidade (SIM); o modelo de Prais-Winsten foi utilizado para análise das tendências.

**Resultados:**

houve 211.658 óbitos no período analisado, com tendência crescente na mortalidade por doença de Alzheimer no país em idosos de 60-69 anos (VPA = 4,3; IC_95%_ 2,9;5,9), 70-79 anos (VPA = 8,1; IC_95%_ 4,8;11,5) e ≥ 80 anos (VPA = 11,3; IC_95%_ 8,1;14,6), e em todas as macrorregiões, faixas etárias e sexo.

**Conclusão:**

o Brasil e todas as suas macrorregiões apresentaram tendência crescente nas taxas de mortalidade por doença de Alzheimer, seguindo a tendência mundial.

## Resumen

**Objetivo:**

analizar las tendencias en las tasas de mortalidad por enfermedad de Alzheimer en Brasil y sus macrorregiones por grupo de edad y sexo, de 2000 a 2019.

**Métodos:**

estudio de series temporales de mortalidad por enfermedad de Alzheimer en Brasil y sus macrorregiones por grupo de edad y sexo; los datos se obtuvieron del Sistema de Información sobre Mortalidad del Ministerio de Salud de Brasil; se utilizó el modelo Prais-Winsten para analizar tendencias.

**Resultados:**

hubo 211.658 óbitos, con tendencia creciente en la mortalidad por enfermedad de Alzheimer en el país, en adultos mayores de 60-69 años (VPA = 4,3; IC_95%_ 2,9;5,9), 70-79 años (VPA = 8,1; IC_95%_: 4,8;11,5) y ≥ 80 años (VPA = 11,3; IC_95%_ 8,1;14,6) y en todas las macrorregiones, grupos de edad y sexo.

**Conclusión:**

Brasil y todas sus macrorregiones mostraron una tendencia creciente en las tasas de mortalidad por enfermedad de Alzheimer siguiendo la tendencia mundial.

**Table:** 

Study contributions	
**Main results**	Brazil and all its macro-regions showed a rising trend in mortality rates due to Alzheimer’s disease, regardless of the stratification performed, in the period from 2000 to 2019.
**Implications for services**	The results found regarding the rising trend of mortality from Alzheimer’s disease in Brazil serve to inform public health policies. Having identified vulnerable groups can guide priority actions in Brazil.
**Perspectives**	In order to move forward in this area, it is essential that public databases are enriched with complete and quality data, which allow more reliable analyses of factors associated with mortality from Alzheimer’s disease in Brazil.

## INTRODUCTION

Alzheimer’s disease is the most common cause of dementia, accounting for 60% to 80% of all cases.^1^ In 2019, dementia affected 55 million people worldwide, and that number is expected to double every 20 years. These projections indicate that there will be 78 million people with dementia in 2030 and 139 million in 2050. In 2019, dementia caused more than one million deaths, being considered the seventh leading cause of death worldwide.^2^ Globally, there is an increase in the prevalence of the disease as age increases. Estimates indicate that the prevalence of the disease is 2% in the 65 to 69 age group, while among people over 90 years old, this index increases to 36%, evidencing the role of aging as a crucial risk factor for the development of Alzheimer’s diease.^2^

Despite the global scenario of increasing prevalence of the disease, the increasing trend in mortality from dementia, in some age groups, has been more pronounced in middle and low-income countries, where two thirds of people living with the disease currently live. This discrepancy between countries indicates that risk factors other than advanced age affect risk of Alzheimer’s disease and dementia, such as lifestyle, vascular disease, psychosocial and environmental context, as well as education and access to health services.^3^

In Brazil, Alzheimer’s disease is a health problem of concern, considering that the population is increasingly aging, together with the increase in mortality rates and the growing prevalence of the disease nationwide in recent years.^4^ ^),(^ ^5^ ^),(^ ^6^ ^)^ There was a 49% increase in the number of deaths from Alzheimer’s disease between 2009 and 2019 in Brazil, making it the seventh leading cause of death.^7^

Despite the seriousness of the problem, there a few training courses for health professionals to care for people with Alzheimer’s disease,^8^ as well as a lack of information, research and bibliographical reviews on the disease nationwide.^9^ This fact is a barrier to the implementation of the Global action plan on the public health response to dementia, established by the United Nations,^10^ which recommends the development, implementation and monitoring of indicators related to dementia at the national level, through records held on health information systems, to improve the availability and quality of data related to the disease. Added to this is the fact that Brazil is heterogeneous in terms of its socioeconomic characteristics and quality of health services. Therefore, in this complex Brazilian context, regional variations in mortality rates due to Alzheimer’s disease are to be expected.

Given this setting, the objective of this study was to analyze trends in mortality rates due to Alzheimer’s disease in Brazil and its macro-regions by age and sex, from 2000 to 2019.

## METHODS

*Design*

This was a time-series study on mortality from Alzheimer’s disease in Brazil and its macro-regions, from 2000 to 2019.

*Context*

Brazil is one of the world’s largest countries, covering an area of 8,515,692.272 km^2^. It is composed of 27 Federative Units and 5,565 municipalities, which are distributed over five macro-regions: North, Northeast, Southeast, South and Midwest.^11^ In 2010, the most populous region was the Southeast, followed by the Northeast, South, North and Midwest, in that order. With regard to the composition of the population according to sex, Brazil has a predominance of females, with males being predominant only in the Northern region. The Brazilian population is in the process of aging, due to the increase in the population aged 65 and over. However, the country’s macro-regions do not follow this pattern. The North and Northeast still show characteristics of a younger population, while the South and Southeast have the most elderly population, and the Midwest shows a similar pattern to Brazil as a whole.

*Participants*

We analyzed the number of deaths due to Alzheimer’s disease - as per code G30 of the International Classification of Diseases and Related Health Problems, Version 10 (ICD-10) ^(^ ^12^ - notified on the Mortality Information System (Sistema de Informação sobre Mortalidade - SIM).

*Variables*

The time series consisted of annual mortality rates due to Alzheimer’s disease, stratified by macro-region, age group (years) and sex. We chose the 60-69, 70-79 and ≥ 80 age groups, for both males and females. Since the mortality rates were calculated by strata, we did not standardize the indicators.^13^ To calculate the rates, the total number of deaths due to Alzheimer’s disease in each age group and sex was divided by the total number of the population at risk, and the quotient was multiplied by 1 million. These rates were calculated for Brazil and its macro-regions, from 2000 to 2019.

*Data sources and measurement*

The number of deaths due to Alzheimer’s disease was obtained from the SIM database, available from the Brazilian National Health System Department of Information Technology (Departamento de Informática do Sistema Único de Saúde - DATASUS) (Ministry of Health).^12^ Population estimates were obtained from the Brazilian Institute of Geography and Statistics (Instituto Brasileiro de Geografia e Estatística - IBGE).^14^ The data were extracted in June 2021.

*Bias control*

In order to control bias, we chose to stratify the study population according to macro-region, age group and sex, with the aim of homogenizing the groups in relation to exposures.

*Study size*

We worked with the number of deaths due to Alzheimer’s disease available on the SIM in June 2021. The period used comprises the years with the most robust data available at the time.

*Statistical methods*

We used the Prais-Winsten^15^ model to perform the trend analysis. The dependent variable considered was the log base 10 transformation of the Alzheimer’s mortality rate, while the independent variable was the year. Annual percentage change (APC) and respective 95% confidence interval (95%CI) were estimated. The trend was considered significant when zero was not part of the APC confidence interval; positive APC indicated a rising trend; and negative APC indicated a falling trend. We used a 5% significance level and we performed the analyses using R software, version 4.1.0.

*Ethical aspects*

The study used secondary public domain data. As such it was exempt from appraisal by a Research Ethics Committee.

## RESULTS

There were 211,658 deaths from Alzheimer’s disease in Brazil between 2000 and 2019, 64% of which were female. Seventy-three percent of the deaths related to people aged 80 or over, 23% were in the 70-79 age group and 4% in the 60-69 age group. The majority of these deaths were recorded in the Southeast region (56%), followed by the Southern (20%), Northeast (16%), Midwest (6%) and Northern (2%) regions.

**Figure 1:**
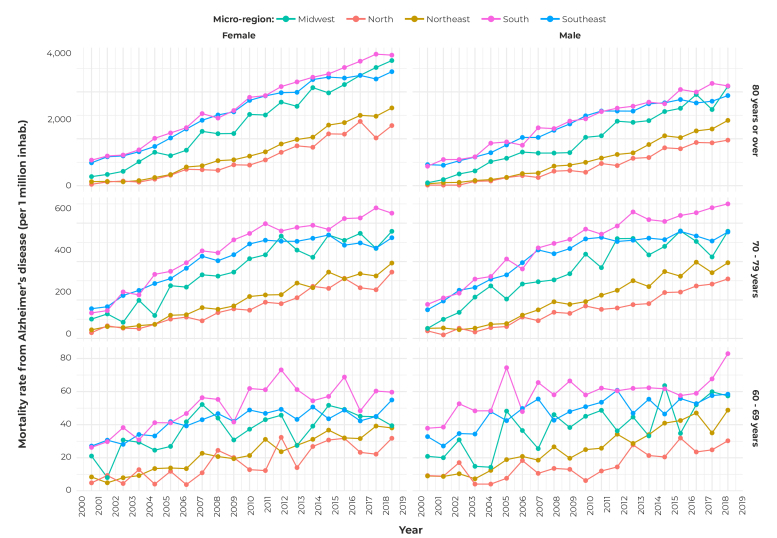
Mortality rate from Alzheimer’s disease (per 1 million inhabitants), by Brazilian region, sex and age, 2000-2019

Figure 1, which presents the Alzheimer’s disease mortality rate time series stratified by Brazilian region, sex and age, during the study period, shows the rise in the mortality rate over time, for all variables analyzed.

**Figure 2:**
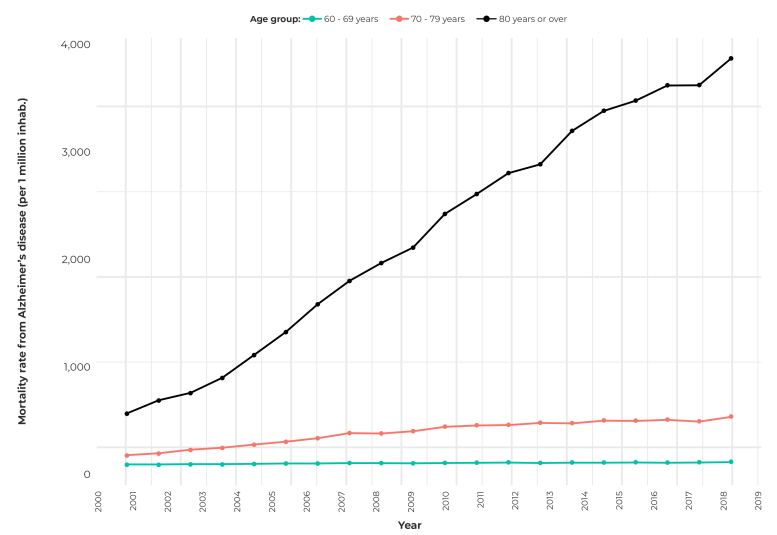
Mortality rate from Alzheimer’s disease (per 1 million inhabitants) in Brazil, by age group, 2000-2019

In turn, Figure 2 shows the upward trend of the mortality rate between the years 2000 and 2019 in Brazil, by age group. It can be seen that the mortality rate in the group of individuals aged 80 years or over was higher than in the other age groups, in the period analyzed.

**Figure 3:**
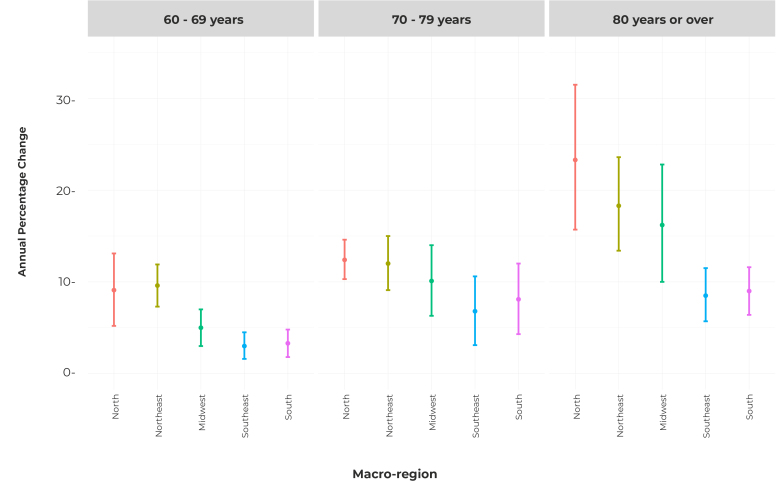
Annual percentage change (APC) and 95% confidence interval (95%CI) of the total mortality rate from Alzheimer’s disease, by macro-region and age group, Brazil, 2000-2019

Table 1 presents the trend analysis and mean values of the Alzheimer’s disease mortality rates, according to place of occurrence, age group and sex. The highest means were found for the group of individuals aged 80 years or older, and the mean female mortality rate was higher when compared to males. The Southern and Southeast regions had the highest mean mortality rates, regardless of the stratification performed, while the Northern and Northeast regions had the lowest means. It was also found that the mean Alzheimer’s disease mortality rates in Brazil, in the period from 2000 to 2019, for males, in the 60-69 (41.4) and 70-79 (356.3 ) age groups, were higher than the mean mortality rates for females, in both age groups (36.8 for the 60-69 age group; and 346.5 for the 70-79 age group). The same happened in most of the Brazilian macro-regions. Regarding the trend analysis, and considering the stratifications performed, Brazil and all its macro-regions showed a statistically significant rising trend during the study period. Furthermore, in the 80 and over age group, APC was higher in the North (APC = 23.3; 95%CI 15.7;31.5; p-value < 0.001), Northeast (APC = 18.3; 95%CI 13.4;23.6; p-value < 0.001) and Midwest (APC = 16.2; 95%CI 10.0;22.8; p-value < 0.001), compared to the South (APC = 9.0; 95%CI 6.4;11.6; p-value < 0.001) and Southeast (APC = 8.5; 95%CI 5.7;11.5; p-value < 0.001) ( Figure 3).

**Table 1:** Mean, annual percentage change (APC) and 95% confidence interval (95%CI) of mortality rates from Alzheimer’s disease (per 1 million inhabitants), by region, sex and age, Brazil, 2000-2019

Macro-region	Age group (years)	Total			Female			Male		
		Mean	APC (95%CI)	p-value^a^	Mean	APC (95%CI)	p-value^a^	Mean	APC (95%CI)	p-value^a^
North	60-69	16.5	9.1 (5.2;13.1)	< 0.001	17.2	10.1 (7.2;13.2)	< 0.001	16.6	7.2 (3.8;10.7)	< 0.001
	70-79	155.5	12.4 (10.3;14.6)	< 0.001	164.9	11.9 (10.0;13.8)	< 0.001	145.7	13.5 (11.0;16.0)	< 0.001
	≥ 80	992.8	23.3 (15.7;31.5)	< 0.001	1,134.9	19.9 (15.1;24.9)	< 0.001	818.2	24.7 (14.9;35.2)	< 0.001
Northeast	60-69	23.9	9.6 (7.3;11.9)	< 0.001	22.3	9.7 (7.3;12.3)	< 0.001	25.7	9.8 (7.7;12.0)	< 0.001
	70-79	199.7	12.0 (9.1;15.0)	< 0.001	198.1	12.0 (9.5;14.5)	< 0.001	201.8	12.2 (8.9;15.7)	< 0.001
	≥ 80	1,298.9	18.3 (13.4;23.6)	< 0.001	1,422.1	17.3 (12.2;22.6)	< 0.001	1,116.0	20.4 (15.0;26.2)	< 0.001
Midwest	60-69	37.5	5.0 (3.0;7.0)	< 0.001	36.6	4.7 (1.6;7.9)	0.005	38.6	5.7 (3.5;8.0)	< 0.001
	70-79	351.7	10.1 (6.3;14.0)	< 0.001	352.9	9.6 (6.7;12.7)	< 0.001	350.3	11.0 (6.3;15.9)	< 0.001
	≥ 80	2,380.3	16.2 (10.0;22.8)	< 0.001	2,700.2	14.3 (9.8;19.1)	< 0.001	1,979.1	19.6 (10.5;29.5)	< 0.001
Southeast	60-69	44.6	3.0 (1.6;4.5)	< 0.001	42.0	3.0 (1.7;4.3)	< 0.001	47.7	3.0 (1.6;4.4)	< 0.001
	70-79	412.9	6.8 (3.1;10.6)	0.001	403.6	6.6 (2.9;10.4)	0.001	425.4	7.1 (3.1;11.2)	0.001
	≥ 80	2,929.7	8.5 (5.7;11.5)	< 0.001	3,163.6	8.8 (5.6;12.1)	< 0.001	2,524.8	8.0 (5.0;11.1)	< 0.001
South	60-69	54.4	3.3 (1.8;4.8)	< 0.001	50.7	4.0 (2.1;6.0)	< 0.001	58.6	2.4 (1.2;3.5)	< 0.001
	70-79	473.8	8.1 (4.3;12.0)	< 0.001	462.7	8.4 (4.4;12.6)	< 0.001	488.3	7.2 (4.4;10.1)	< 0.001
	≥ 80	3,145.9	9.0 (6.4;11.6)	< 0.001	3,413.2	9.0 (6.4;11.7)	< 0.001	2,690.6	8.7 (6.6;10.8)	< 0.001
Brazil	60-69	38.9	4.3 (2.9;5.8)	< 0.001	36.8	4.5 (2.8;6.1)	< 0.001	41.4	4.2 (3.0;5.4)	< 0.001
	70-79	350.8	8.1 (4.8;11.5)	< 0.001	346.5	8.0 (4.6;11.6)	< 0.001	356.3	8.2 (4.9;11.6)	< 0.001
	≥ 80	2,368.1	11.3 (8.1;14.6)	< 0.001	2,609.1	11.1 (7.7;14.5)	< 0.001	1,985.4	11.7 (8.5;15.0)	< 0.001

a) Prais-Winsten model.

## DISCUSSION

Mortality due to Alzheimer’s disease was found to be rising in Brazil and its macro-regions between 2000 and 2019. The Northern and Northeast regions showed greater annual percentage change, compared to the Southern and Southeast regions, in octogenarians. Mean mortality rates due to Alzheimer’s disease were high in the 80 and over age group and also in females in this age group. However, the mean mortality rates were higher for males in the 60-69 and 70-79 age groups. In addition, the Northeast and Northern regions had lower mean Alzheimer’s disease mortality rates in relation to the other macro-regions, regardless of age group.

The results found are in line with a study that showed an increase in mortality rates due to Alzheimer’s disease in Brazil, from 2010 to 2019.^16^ The higher mortality rate APCs observed in the Northern and Northeast regions can be explained by the fact that, over time, there was a greater decrease in the number of deaths from ill-defined causes in these regions, compared to the others - deaths from ill-defined causes fell by 17% and 24%, respectively, in the Northern and Northeast regions, between 2000 and 2019, while they fell by 7%, 5% and 3% in the Midwest, Southeast and Southern regions, respectively. This may mean that there was an improvement in diagnosis of death from Alzheimer’s disease over time in the Northern and Northeast regions, reflected in their greater annual percentage change in the mortality rate.

The findings corroborate a global study that showed higher prevalence of dementia among the elderly and females.^2^ These results also confirm an epidemiological study of Alzheimer’s disease mortality in Brazil between 2010 and 2019, which pointed to a higher percentage of deaths among people aged 80 or over and among females.^16^ In this context, our finding a higher mean mortality rate related to the over 80 age group and the female sex confirms aging and being female as risk factors for the development of Alzheimer’s disease.^2^ ^),(^ ^17^ However, the higher mean mortality rates due to Alzheimer’s disease in males in the 60-69 and 70-79 age groups may be associated with lower life expectancy and a higher number of comorbidities.^18^

The lower mortality rates found in the Northern and Northeast regions, compared to the other regions, can possibly be explained by the high percentage of deaths from ill-defined causes in these regions. During the study period, while mean deaths from ill-defined causes accounted 15% and 14% of deaths, respectively, in the Northern and Northeast regions, deaths from ill-defined causes accounted for 5% in the Midwest, 5% in the South and 8% in the Southeast, suggesting that undiagnosed deaths due to Alzheimer’s disease may occur more in the Northern and Northeast macro-regions. Another possible explanation for the lower means having occurred in these two regions relates to the differences in life expectancy between the Brazilian regions. Between 2000 and 2019, mean life expectancy in the North and Northeast was 71 years, while it was 74 years in the Midwest, 75 years in the Southeast and 76 years in the South.^19^ This is a relevant factor, since prevalence of Alzheimer’s disease increases with age. In the United States, in 2020, for example, prevalence of Alzheimer’s disease in the population aged between 65 and 74 years was estimated at 17% and 47%, respectively, among those between 75 and 84 years old.^1^ Thus, a plausible hypothesis is that the lower mean life expectancy in the Northern and Northeast regions could be reflected in the Alzheimer’s disease mortality rates, since individuals from these regions may not survive long enough to develop the disease.

Other modifiable risk factors associated with Alzheimer’s disease and dementia may also play a critical role in the results. It is estimated that well-established risk factors such as level of education, hearing loss, traumatic brain injury, hypertension, alcohol consumption, obesity, smoking, depression, social isolation, diabetes and air pollution are related to 40% of cases of dementia worldwide.^3^ The potential for prevention is greater in middle and low-income countries, where cases of dementia are growing more sharply.^3^ Access to education, health services and public health policies on dementia prevention could change the scenario of Alzheimer’s disease in Brazil.

The quality of the data is questionable, due to the high percentage of deaths with underlying causes classified as nonspecific or incomplete (garbage codes), which may result in a limitation of this study.^20^ These codes are used for causes that are not the underlying causes of death, or for unspecific causes, which, therefore, hinders identification, development and improvement of public health actions. Moreover, the proportion of deaths from ill-defined causes is a reflection of inequality in access to health and medical care provided to the population.^18^

Despite its limitations, this study used data on the whole of Brazil, collected over a long period of time. Considering Brazil’s vast geographic area and population size, an ecological approach makes it possible to quickly identify the existence of vulnerable groups that need priority actions. In this way, we believe that this study provides important analysis, previously scarce, on Alzheimer’s disease in Brazil.

In conclusion, Brazil and all its macro-regions showed a rising trend in Alzheimer’s disease mortality rates, regardless of sex and age group, following the global trend.
